# Relapsed acute promyelocytic leukemia in a hemodialysis-dependent patient treated with arsenic trioxide: a case report

**DOI:** 10.1186/1752-1947-6-355

**Published:** 2012-10-18

**Authors:** Gregory S Emmons, Richard H Steingart, James A Stewart, Wilson C Mertens

**Affiliations:** 1 Baystate Regional Cancer Program, 3400A Main Street, Springfield, MA 01199, USA; 2 Tufts University School of Medicine, Boston, MA, USA

## Abstract

**Introduction:**

In the relapsed setting, arsenic trioxide remains the backbone of treatment. Scant literature exists regarding treatment of relapsed acute promyelocytic leukemia in patients with renal failure. To the best of our knowledge we are the first to report a safe and effective means of treatment for relapsed acute promyelocytic leukemia in the setting of advanced renal failure, employing titration of arsenic trioxide based on clinical parameters rather than arsenic trioxide levels.

**Case presentation:**

A 33-year-old Caucasian man with a history of acute promyelocytic leukemia in remission for 3 years, as well as dialysis-dependent chronic renal failure secondary to a solitary kidney and focal segmental glomerulosclerosis and human immunodeficiency virus infection, receiving highly active antiretroviral therapy presented to our hospital with bone marrow biopsy-confirmed relapsed acute promyelocytic leukemia. Arsenic trioxide was begun at a low dose with dose escalation based only on side effect profile monitoring and not laboratory testing for induction as well as maintenance without undue toxicity. Our patient achieved and remains in complete hematologic and molecular remission as of this writing.

**Conclusion:**

Arsenic trioxide can be used safely and effectively to treat acute promyelocytic leukemia in patients with advanced renal failure using careful monitoring of side effects rather than blood levels of arsenic to guide therapeutic dosing.

## Introduction

Arsenic trioxide has been used as a treatment for leukemia since the late 1800s [1]. Since that time additional studies have been reported examining the efficacy of arsenic trioxide in relapsed acute promyelocytic leukemia (APL) [2-5], but little has been published concerning the use of this agent in patients with renal failure. Only one case report has been published reporting on the dosing, effectiveness and tolerability of arsenic trioxide therapy in a patient requiring temporary hemodialysis [6]. We report a case of a patient with end-stage chronic renal failure on daily home hemodialysis successfully treated with curative intent arsenic trioxide for relapsed APL without employing laboratory monitoring of arsenic trioxide levels during the induction, consolidation and maintenance phases of treatment in relapsed APL.

## Case presentation

A 33-year-old Caucasian man presented to an outside hospital 6 years ago with pancytopenia. Comorbid conditions consisted of human immunodeficiency virus infection diagnosed 19 years ago and treated with highly active antiretroviral therapy, and an 8-year history of chronic kidney disease secondary to focal segmental glomerulosclerosis and congenital solitary kidney. Bone marrow at presentation was notable for 75% involvement with abnormal promyelocytes. Fluorescence *in-situ* hybridization studies and reverse transcriptase-polymerase chain reaction (RT-PCR) confirmed the t(15;17) translocation and presence of the promyelocytic leukemia *(PML)-RARα* fusion transcript respectively; a diagnosis of APL was made. Induction therapy consisted of daunorubicin 45mg/m^2^ daily on days 1 through to 3 with cytarabine 100mg/m^2^ daily on days 1 through to 7 concurrent with all-trans retinoic acid (ATRA) 40mg/m^2^ administered twice daily. Two cycles of consolidation daunorubicin 45mg/m^2^ daily on days 1 through to 3 were subsequently administered followed by 4 months of maintenance ATRA therapy; ATRA was discontinued prematurely due to near lethal pancreatitis in the setting of marked hypertriglyceridemia associated with the drug. He achieved and remained in remission, confirmed by repeated RT-PCR, for 3 years.

He presented subsequently to our hospital with neutropenic fever, pancytopenia, and low-grade disseminated intravascular coagulation. Bone marrow aspiration demonstrated relapsed APL with 80 to 90% cellularity and promyelocytes compromising 66% of the differential leukocyte count, as well as the t(15;17)(q22;q12) translocation and *PML-RARα* fusion transcript.

Given the patient’s history of severe pancreatitis related to ATRA, induction consisted of single agent arsenic trioxide 0.1mg/kg IV every other day while receiving hemodialysis (commenced 6 months prior to the diagnosis of his relapse) four times weekly. Complete blood counts, sodium, potassium, chloride, bicarbonate, blood urea nitrogen, creatinine, glucose prothrombin time, activated prothrombin time, and fibrinogen were monitored daily. The QTc duration was monitored daily via an electrocardiogram as well as pre- and post-dialysis on dialysis days for the first 2 weeks of induction. Given an observed stability of the QTc, electrocardiograms were then monitored weekly. Magnesium and potassium levels were maintained above 2.0mEq/L and 4.0mmol/L respectively. Aspartate aminotransferase and alanine aminotransferase levels were monitored weekly. Following 60 days of induction, a persistently elevated *PML-RARα* fusion transcript was detected on RT-PCR. The dose of arsenic trioxide was then increased to 0.15mg/kg every other day and continued for an additional 60 days. During this interval the patient required a brief hospital stay and arsenic trioxide dose interruption for hyperglycemia, which was determined to be adult-onset insulin-dependent diabetes probably exacerbated by arsenic trioxide therapy. A bone marrow biopsy after the second 60-day period confirmed remission. Consolidation consisted of single agent idarubicin at 12mg/m^2^ on days 1 and 2 every 28 days for two cycles. Maintenance consisted of 27 doses of arsenic trioxide. Treatment commenced with 0.1mg/kg arsenic trioxide Monday, Wednesday, and Friday; excessive fatigue after nine doses prompted the remaining doses to be delivered on a Tuesday and Friday schedule.

The patient has moved from the area but remains alive and free of relapse at the time of this writing. Given his comorbidities, the patient was not felt to be a candidate for high-dose chemotherapy with stem cell support.

## Discussion

There is a paucity of literature involving treatment of relapsed APL in the setting of advanced renal failure requiring dialysis and the need for surveillance of arsenic levels during the treatment process. Two cases involving the successful usage of arsenic trioxide in dialysis-dependent patients have been published to date: one involving a patient receiving hemodialysis thrice weekly whereas the other received arsenic trioxide in the setting of continuous ambulatory peritoneal dialysis; both employed serum arsenic levels [6,7].

Arsenic trioxide is rapidly cleared from blood; yet, only a small fraction of the daily drug dose undergoes urinary excretion, making therapy in patients with impaired renal function potentially challenging. The majority of the agent is sequestered in bodily tissues and undergoes delayed elimination over many weeks to months [8,9], suggesting that excess accumulation in the serum of patients with impaired renal function is unlikely. Readily available assays measure only elemental arsenic, and although newer and more sensitive assays appropriately measure organic arsenical compounds, these are not accessible in a timely fashion. Dialysis-induced compartmental shifts further limit the value of accurate measurement of arsenic trioxide; in the treatment of toxic arsenic ingestion, post-dialysis levels may actually exceed pre-dialysis levels [10,11].

In non-dialysis-dependent patients, arsenic trioxide has demonstrated efficacy during induction employing both fixed daily dosing (10mg or 15mg daily) and weight-based dosing (0.06–0.20mg/kg/day) [2-5]. We adopted a ‘go low, go slow’ approach in the treatment of our patient, beginning with 0.1mg/kg arsenic trioxide every other day while monitoring for common side effects including: QTc prolongation, hepatic dysfunction, fluid retention, electrolyte imbalances, cytopenias and constitutional changes. As arsenic trioxide concentrations were not assessed or monitored, the titration of arsenic trioxide was based on toxicity profile. Based on the pharmacodynamics noted above, we suspected that residual arsenic, having accumulated in various bodily tissues, would continue to provide efficacy long after our patient’s last dosing. Consequently, consolidation consisted of single agent idarubicin followed by maintenance arsenic trioxide. Surveillance for the *PML-RARα* fusion transcript via RT-PCR continues to suggest remission at the time of this writing.

## Conclusion

Arsenic trioxide can be used safely and effectively to treat APL in patients with advanced renal failure. Given the difficulty in obtaining accurate serum levels of arsenic trioxide and the relative ease of monitoring for common treatment-related side effects, we suggest foregoing monitoring of arsenic trioxide levels during treatment of relapsed APL in dialysis-dependent patients. Combining a low initial dose with slow dose escalation with vigilant monitoring for common side effects provides an effective and efficient means of administering arsenic trioxide therapy.

## Consent

Written informed consent was obtained from the patient for publication of this case report. A copy of the written consent is available for review by the Editor-in-Chief of this journal.

## Competing interests

The authors declare that they have no competing interests.

## Authors’ contributions

GSE and RHS cared for the patient and performed the histological evaluations of the bone marrow assessments, and interpreted the clinical laboratory data. GSE and WCM performed the literature review and wrote the manuscript; GSE, WCM, and JAS were major contributors in writing the manuscript. All authors read and approved the final manuscript.
